# Prevalence of gambling problems, help-seeking, and relationships with trauma in veterans

**DOI:** 10.1371/journal.pone.0268346

**Published:** 2022-05-25

**Authors:** Olivia Metcalf, Ellie Lawrence-Wood, Jenelle Baur, Miranda Van Hooff, David Forbes, Meaghan O’Donnell, Nicole Sadler, Stephanie Hodson, Helen Benassi, Tracey Varker, Malcolm Battersby, Alexander C. McFarlane, Sean Cowlishaw

**Affiliations:** 1 Department of Psychiatry, Phoenix Australia–Centre for Posttraumatic Mental Health, The University of Melbourne, Melbourne, Australia; 2 Military and Emergency Services Health Australia, The Hospital Research Foundation, Adelaide, Australia; 3 Department of Veterans’ Affairs, Open Arms–Veteran & Families Counselling, Canberra, Australia; 4 Australian Department of Defence, Joint Health Command, Joint Capabilities Group, Carlton, Australia; 5 College of Medicine and Public Health, Flinders University, Adelaide, Australia; 6 Population Health Sciences, Bristol Medical School, University of Bristol, Bristol, United Kingdom; University of São Paulo, BRAZIL

## Abstract

**Background and aims:**

Veterans who have recently left the military (i.e., transitioned) may be vulnerable to the development of psychiatric disorders, but little is known about gambling problems in this population. This study investigated the prevalence and risk factors of gambling problems, help-seeking amongst veterans with gambling problems, and relationships with trauma and posttraumatic psychopathology.

**Methods:**

Cross-sectional self-report survey data from 3,511 Australian Defence Force members who left the military within the past five years. Surveys included measures of gambling problems (PGSI); depressive symptoms (PHQ-9); posttraumatic stress disorder (PCL-5); help-seeking behaviours; military and non-military-related trauma.

**Results:**

Prevalence rates for problem gambling (PGSI ≥ 5) were 4.6%, while an additional 8.8% were classified in terms of at-risk gambling (PGSI = 1–4). Time since leaving the military was not associated with gambling problems. Only 2.1% of veterans with problem gambling reported help-seeking for their gambling. While trauma exposure, depression, and Posttraumatic Stress Disorder (PTSD) were all related to gambling problems at the bivariate level, only arousal and dysphoric-related affect were uniquely associated with gambling problems when adjusting for covariates.

**Discussion:**

Gambling problems may be under-recognised relative to other psychiatric issues. Posttraumatic mental health problems, rather than trauma exposure per se, may explain the relationship between trauma and gambling problems.

**Conclusions:**

Some veterans are in a period of vulnerability during transition out of military service, and harms associated with gambling problems may be exacerbated during this period.

## Introduction

The transition out of military service can be a time of significant change and challenge for veterans. Research has highlighted increasing vulnerability to psychiatric problems with time since transition, and evidence indicates that risk may double over the first five years [1]. Veterans may also take with them into civilian life the cultures, beliefs, and behaviours relating to help-seeking and mental health that develop in military service, and some of these may subsequently compound or exacerbate psychiatric disorders and other mental health problems [2]. Although not all veterans experience challenges, some can face psychosocial and financial challenges during transition which may also be related to psychiatric conditions [3].

There is emerging research which indicates that gambling problems are an under recognised issues in veteran and military populations. A comprehensive study of Australian military personnel who had recently returned from deployment found rates of 2.0% for past year problem gambling, with an additional 5.7% classified in terms of at-risk gambling (i.e., 7.7% of participants reported any gambling problems) [4]. Very little research has been conducted on gambling problems in veteran populations; that is, once an individual leaves the military. Internationally, US research suggests rates of 2.2–4.2% for past year problem gambling in veteran populations [5, 6], and lifetime prevalence rates around 10% [7]. UK research has identified rates of 1.4%, and 3.6% lifetime problem gambling and at-risk gambling, respectively [8]. A small Australian study found that more than a quarter (28%) of veterans in treatment for posttraumatic stress disorder (PTSD) reported probable problem gambling [9], but more recent comprehensive studies are needed.

Given that rates of psychiatric disorders often increase for populations that have left the military [1], there is reason to suspect this includes risk of gambling problems. The highly stressful nature of transition for some veterans, and risk-taking behaviour associated with PTSD may make veterans particularly vulnerable to the development of gambling problems, which may also occur in the context of changes to their financial, social, community, and housing situations, and potentially feelings of isolation from civilian society. Gambling behaviours are often used as a form of socialisation, both with civilians and other veterans, and this may be exacerbated by the sense of disconnection veterans can feel during transition [8]. In addition, veterans face real barriers to help-seeking for psychiatric disorders and other mental health issues, due to both stigma associated with mental health problems, delays in treatment engagement, and other real-world barriers to care [10]. Understanding the nature of help-seeking in veterans with gambling problems is thus needed to inform strategies for enhancing early access and engagement in treatment.

Increased rates of psychiatric disorders may be due in part to the high rates of trauma exposure in veteran populations, and attention has turned to how trauma and gambling problems may also interact [11]. Research indicates high rates of trauma amongst civilian problem gamblers [12], and suggests that these exposures may create vulnerabilities to the development and maintenance of gambling problems [13]. Major theoretical models of gambling problems posit that adverse or traumatic experiences, combined with a strong desire to alter arousal and/or mood states, can lead to the development of addictive problems [14]. Although complex and differentiated across types of gamblers and gambling behaviours, overall, research shows that gambling can alter physiological and self-reported arousal, and that these alterations may underpin the development and maintenance of gambling problems [15]. Strongly linked to arousal is the tendency for gamblers to gamble in order to escape or manage dysphoria or negative mood states [12].

Military and veteran populations are at risk of exposure to traumatic events, both through deployment and other service-related traumatic experiences, as well as pre-military trauma [16]. Previous research in military personnel has shown that PTSD was associated with gambling behaviours, but that stressful pre-deployment, combat, or post-battle experiences were not [6]. Research has not used measures of traumatic exposure and gambling problems in military and veteran populations to consider mechanisms by which trauma exposure may influence gambling problems. Given the emerging evidence of gambling problems in veteran and military populations, it is important to understand how trauma, and symptoms of associated posttraumatic psychiatric disorders, including PTSD and depression, relate to gambling problems, as well as help-seeking, in order to more effectively prevent and treat gambling problems in veterans.

The aims of this study were to investigate:

1. The prevalence of gambling problems in a representative sample of Australian veterans who have recently left the military, and vulnerable sub-groups;
2. Patterns of help-seeking amongst veterans with gambling problems; and
3. The role of trauma exposure and posttraumatic mental health problems in gambling problems in a veteran population.

## Method

### Participants and procedure

Data was drawn from 3,511 Australian Defence Force (ADF) members who had left the military within the past five years. The sample was predominately male (84.3%), and aged between 28–47 years (54.3%). Half the sample served in the Army (56.1%), with 19.9% serving in the Navy, and 24.0% serving in the Air Force. A significant minority (40.2%) reported serving more than 20 years.

Cross-sectional survey data from the Transition and Wellbeing Research Programme (The Programme) was used, from which 23,974 transitioned ADF members were invited to complete a self-report survey, and responses were received from n = 4,326 (18.0% response rate) [1]. The current analytic sample comprised n = 3,511 transitioned ADF members who were classified as survey responders to relevant sections of the questionnaire that contained questions of interest (n = 4,165). The sampling frame comprised the Military and Veteran Research Study Roll, which was derived for The Programme from a range of data sources, including administrative records from the Department of Defence, and contact data from Department of Veterans’ Affairs (DVA). These records were cross-referenced against the National Death Index. An email was sent to 23,974 transitioned ADF members inviting them to complete a 60-minute online self-report survey. Participants could also opt to have a hardcopy survey posted to them.

### Measures

**Past year gambling problems** were measured using the Problem Gambling Severity Index [PGSI; 17] The PGSI comprises 9-items which are scored on a 4-point scale (0 = never, 3 = almost always), and have high internal consistency (Cronbach’s α = 0.90) and strong associations with comparable measures [18]. The current study used a criterion of PGSI ≥5 for problem gambling, which has been shown to yield greatest classification accuracy relative to clinician ratings involving detailed case conceptualisations, while scores of PGSI 1–4 indicated at-risk gambling, consistent with prior research [18–20]. Any gambling problems was defined as 1+ on the PGSI.

**Depression** symptoms were assessed using the 9-item Patient Health Questionnaire (PHQ-9). Each item comprising the PHQ-9 evaluates the frequency of one of the nine Diagnostic and Statistical Manual of Mental Disorders (DSM-IV) criteria for major depression over the past two weeks. The PHQ-9 has been validated against clinical diagnoses from medical professionals and has superior operating characteristics to alternative scales [21]. A summed score of ≥ 10 on the PHQ-9 was used to identify probable major depressive episode in this study [21].

**Posttraumatic Stress Disorder (PTSD)** symptoms were assessed using the Posttraumatic Stress Disorder checklist for DSM-5 [PCL-5; 22] a 20-item self-administered measure asking about symptoms in the past month, on a scale from 0 ‘not at all’ to 4 ‘extremely’. Items were summed to give a total severity score ranging from 0 to 80. Consistent with previous literature, scores 33+ were used to indicate a probable PTSD case [23]. Additionally, items of each of the 4 PCL-5 subscales were summed to give total severity scores for each; 5 re-experiencing items (cluster B), 2 avoidance items (cluster C), 7 negative cognitions and mood items (cluster D), and 6 arousal items (cluster E).

**Lifetime non-military trauma exposure** was assessed using a list of 24 traumatic events taken from the Composite International Diagnostic Interview (CIDI 3.0) PTSD module [24]. Participants were asked if they had ever experienced any of the events in their lifetime. Examples included ‘being in a life-threatening automobile accident’, and ‘having someone close to you die unexpectedly’. The number of events reported were summed to give an indication of total lifetime military trauma. Two of the original 26 CIDI events: ‘combat (military or organised non-military group)’ and ‘peacekeeper or relief worker in a war zone or in a place where there was ongoing terror of people because of political, ethnic, religious or other conflicts’ were excluded as they relate to military trauma.

**Traumatic deployment exposures** was assessed using items taken from a previous study [25]. Participants reported how many times they had experienced a list of 12 deployment exposures during their military career. Response categories ranged from ‘Never’ to ‘10+ times’. Examples of events included ‘discharge of weapon in direct combat’ and ‘handled or saw dead bodies’. The number of events reported were summed to give an indication of career traumatic deployment exposure.

**Lifetime concern about mental health** was assessed by asking participants whether they have ever been concerned about their mental health (e.g., stress, anxiety, depression, anger, relationship problems).

**Assistance for mental health** was assessed by asking participants whether they had ever had assistance for their mental health. They could choose multiple responses from: ‘No’, ‘Yes, more than 12 months ago’, ‘Yes, in the last 12 months’, and ‘Yes, currently’ [27].

**Reasons for seeking help** was assessed by asking participants to indicate what primary reason and secondary reason(s) led them to seeking care. Examples included ‘anger’, ‘depression’, and ‘gambling’.

### Statistical analyses

Data-file management and preliminary exploration were conducted using SPSS version 25, while MPlus (version 8) was used subsequently for substantive analyses. Preliminary analyses involved quantification of the extent of missing data (e.g., due to item non-response), production of descriptive statistics to characterise the sample, and a descriptive analysis of help-seeking. Aggregate scale scores for the PGSI were formed and defined using cut-off criteria that distinguished gambling problems across a continuum of severity. Prevalence point estimates (and 95% Confidence Intervals; CIs) were thus calculated to indicate levels of (a) at-risk gambling (PGSI = 1–4) and (b) problem gambling (PGSI ≥ 5). These prevalence estimates were considered relative to the estimated rates of other psychiatric disorders (e.g., depression) and substance use problems (e.g., harmful drinking). Mean imputation of data occurred on PHQ-9 and PCL-5 for cases with ≤ 25% missing data on that measure. This equated to imputation of up to 4 items on the PCL-5. Up to 1 missing value was imputed for the PGSI.

For prevalence data, weighted prevalence estimates are presented, with the methods for creating weights described elsewhere [1]. All survey data was weighted using distinct strata for sex, Service (Navy, Army, or Air Force), rank, and medical fitness. The remaining analyses used unweighted data. To explore sub-groups at-risk of gambling problems, a series of multinomial logistic regression models were estimated to examine the socio-demographic and service-related risk factors for gambling problems. For these analyses, a 3-level measure of gambling problems (no gambling risk = 0; at-risk gambling: PGSI = 1–4; problem gambling: PGSI ≥ 5) was specified as the outcome (dependent) variable. This measure was regressed on socio-demographic and service-related characteristics that were explanatory variables. To explore the relationships with gambling problems and trauma, a stepped negative binomial regression model examining predictors of gambling problems (PGSI total score) was conducted. Demographic and service characteristics (age, sex, relationship status, education, rank, serving status and ever deployed) were entered as covariates in step 1. Number of non-military lifetime traumas and number of traumatic deployment experiences were entered in step 2. The depression (PHQ-9) total score was entered in step 3. The sum of items on the four PCL-5 subscales were then entered in step 4. Adjusted Incidence Rate Ratios (IRR) ‘s and 95% Confidence Intervals (CIs) were calculated. IRR is calculated as the ratio of two incidence rates. An IRR is the estimated rate ratio for a given variable, if the other variables in the model are held constant.

### Ethics

This study was granted ethical approval by the Departments of Defence and Veterans’ Affairs Human Research Ethics Committee (Protocol number E014-018).

## Results

The weighted prevalence of problem gambling was 4.6% among recently transitioned veterans, with a further 8.8% of these ex-serving members reporting at-risk gambling. Thus, there was a combined total of 13.4% (95% CI = 13.0% - 13.8%) of transitioned members that reported any gambling problems (see Table 1). Regression analyses indicated that the highest rate of at-risk gambling was observed among younger adults aged 18–27 years (11.2%), with lower rates observed among the older age groups. Males were more likely to report both at-risk gambling and problem gambling when compared to females. Being unemployed (including those on a disability support pension) was associated with higher levels of both at-risk gambling and problem gambling, when compared to respondents who were in full or part-time paid work. However, there were no discernible links between gambling problems and main source of income.

**Table 1:** Weighted gambling problems, depression, and PTSD rates (n = 3,511).

	n	%	95% CI	
			LB	UB
Gambling problems (past-year)				
At-risk gambling	2174	8.8	8.4	9.1
Problem gambling	1153	4.6	4.4	4.9
Any gambling problems	3327	13.4	13.0	13.8
Depression (past 2 weeks)	6246	30.7	30.3	31.6
PTSD (past-month)	4044	19.1	18.5	19.6

Table 2 shows findings from multinomial logistic regression analyses examining the service-related risk factors gambling problems. As can be seen, veterans who reported Army service reported higher levels of at-risk gambling and problem gambling when compared to Air Force personnel, while increased rates of both at-risk and problem gambling were also reported by Non-commissioned Officers(NCOs)s / Other Ranks (when compared to Commissioned Officers), and among ex-serving members (when compared to Active or Inactive Reservists). Veterans who were medically discharged also reported higher rates of at-risk and problem gambling, while those who reported histories of operational deployment reported increased rates of at-risk gambling when compared to those who had never deployed. Department of Veterans’ Affairs (DVA) clients and those receiving DVA treatment support since transition were more likely to report both at-risk and problem gambling compared to non-DVA clients and those not receiving DVA treatment support. In contrast, gambling problems did not differ by years of service and time since transition. Table 3 presents help-seeking rates and reasons amongst veterans with gambling problems, indicates that specific help-seeking for gambling was uncommon in this sample.

**Table 2:** Multinomial logistic regression models indicating socio-demographic predictors of at risk and problem gambling.

	No gambling problems (n = 3091)		At risk gambling (n = 286)	Problem gambling (n = 134)		Logistic regression models							
	(0)		(1)	(2)		(0) vs (1)					(0) vs (2)		
	n	%	n	%	n	%	OR	LB	UB	OR		LB	UB
Age group													
18–27	282	82.9	38	11.2	20	5.9	ref			ref			
28–37	858	87.7	83	8.5	37	3.8	0.72	0.48	1.08	0.61		0.35	1.07
38–47	807	88.6	70	7.7	34	3.7	0.64*	0.42	0.98	0.59		0.34	1.05
48–57	679	89.9	51	6.8	25	3.3	0.56*	0.36	0.87	0.52*		0.28	0.95
58+	436	87.7	43	8.7	18	3.6	0.73	0.46	1.16	0.58		0.30	1.12
Sex													
Male	2562	86.6	267	9.0	131	4.4	2.90***	1.81	4.66	9.02***		2.86	28.43
Female	529	96.0	19	3.4	3	0.5	ref			ref			
Employment status													
Full/ part time paid work	2087	88.8	185	7.9	79	3.4	ref			ref			
Unemployed (incl. disability support pension)	420	83.8	54	10.8	27	5.4	1.45*	1.05	2.00	1.70*		1.08	2.66
Retired	293	85.4	32	9.3	18	5.2	1.23	0.83	1.83	1.62		0.96	2.75
Other (student, unpaid work)	262	91.9	13	4.6	10	3.5	0.56*	0.31	1.00	1.01		0.52	1.97
Main source of income													
Wage/salary/own business/partnership	1845	88.7	163	7.8	71	3.4	ref			ref			
Age pension or Superannuation	522	87.6	54	9.1	20	3.4	1.17	0.85	1.62	1.00		0.60	1.65
Invalidity service pension or VEA/SRCA/MRCA compensation	312	85.2	34	9.3	20	5.5	1.23	0.84	1.82	1.67		1.00	2.78
Other	371	87.1	33	7.7	22	5.2	1.01	0.68	1.49	1.54		0.94	2.52
Service													
Army	1704	86.5	176	8.9	91	4.6	ref			ref			
Navy	618	88.4	62	8.9	19	2.7	0.97	0.72	1.32	0.58*		0.35	0.95
Air Force	769	91.4	48	5.7	24	2.9	0.60**	0.43	0.84	0.58*		0.37	0.92
Rank													
Commissioned Officer	1018	93.1	56	5.1	20	1.8	ref			ref			
Non-Commissioned Officer / Other Ranks	2073	85.8	230	9.5	114	4.7	2.02***	1.49	2.73	2.80***		1.73	4.53
Time served in Regular ADF													
0–4 years	339	87.4	28	7.2	21	5.4	0.91	0.59	1.40	0.658		1.59	0.94
5–9 years	679	86.4	77	9.8	30	3.8	1.25	0.92	1.69	0.158		1.13	0.71
10–19 years	793	88.9	67	7.5	32	3.6	0.93	0.68	1.27	0.642		1.03	0.66
20+ years	1230	88.5	112	8.1	48	3.5	ref					ref	
Years since transition													
0	272	88.0	28	9.1	9	2.9	1.04	0.62	1.73	0.90		0.39	2.12
1	618	88.4	48	6.9	33	4.7	0.78	0.50	1.22	1.46		0.77	2.76
2	605	90.2	49	7.3	17	2.5	0.81	0.52	1.27	0.77		0.37	1.57
3	609	86.3	64	9.1	33	4.7	1.06	0.69	1.61	1.48		0.78	2.80
4	472	87.7	45	8.4	21	3.9	0.96	0.61	1.51	1.21		0.61	2.42
5+	382	88.0	38	8.8	14	3.2	ref			ref			
Medical discharge													
No	2446	89.1	212	7.7	87	3.2	ref			ref			
Yes	613	83.9	73	10.0	45	6.2	1.37*	1.04	1.82	2.06***		1.43	2.99
Ever deployed													
No	594	91.5	37	5.7	18	2.8	ref			ref			
Yes	2495	87.2	249	8.7	116	4.1	1.60*	1.12	2.29	1.53		0.93	2.54
Veterans Affairs connected													
No	1245	91.2	86	6.3	34	2.5	ref			ref			
Yes	1496	85.3	170	9.7	87	5.0	1.65***	1.26	2.16	2.13***		1.42	3.19

Note:

*** = p < 0.001

** = p < 0.01

* = p < 0.05.

**Table 3:** Primary reasons for seeking care by gambling status.

	No gambling problems		At risk gambling		Problem gambling	
	(n = 3091)		(n = 286)		(n = 134)	
	n	%	n	%	n	%
Anger	186	11.2	21	12.4	12	12.8
Anxiety or depression	750	45.3	71	41.8	38	40.4
Functional impairment (relationship or work problems)	380	22.9	28	16.5	17	18.1
Sleep or nightmares	123	7.4	21	12.4	11	11.7
Alcohol or other drug problems	38	2.3	7	4.1	2	2.1
Gambling	0	0.0	0	0.0	2	2.1
Other (including pain)	180	10.9	22	12.9	12	12.8

Greater numbers of military and non-military (lifetime) traumatic events were both associated with rates of at-risk gambling and problem gambling, while symptoms of PTSD were also significantly associated with any gambling problems (Table 4). The number of lifetime non-military traumatic events was also a significant predictor of gambling problems when controlling for socio-demographic and service-related characteristics. In contrast, the number of military-related trauma exposures was not significantly associated with gambling problems when controlling for lifetime trauma history. PHQ-9 depression scores were related to gambling problems when entered into the model in step 2, while the arousal symptoms of PTSD was a significant unique predictor in step 3. Both depression and arousal scores were significant predictors of gambling problems in the final model (step 3), while non-military traumatic events were no longer significant when controlling for these post-traumatic mental health problems (Table 5).

**Table 4:** Multinomial logistic regression models indicating PTSD and trauma as predictors of gambling problems.

	No gambling problems		At risk gambling		Problem gambling		Logistic regression models								
	(n = 3091)		(n = 286)		(n = 134)										
	(0)		(1)		(2)		(0) vs (1)			(0) vs (2)			(1) vs (2)		
	n	%	n	%	n	%	OR	LB	UB	OR	LB	UB	OR	LB	UB
Trauma exposures															
Non-military traumatic events (M, SD)	2.6	(2.6)	3.4	(2.9)	3.6	(3.3)	1.10***	1.05	1.14	1.12***	1.06	1.19	1.03	0.96	1.09
Military-related trauma exposures (M, SD)	4.0	(3.8)	5.4	(4.0)	5.3	(4.0)	1.10***	1.06	1.13	1.09***	1.04	1.13	0.99	0.94	1.04
PCL-5 PTSD subscales															
Re-experiencing (B)	3.1	(4.7)	4.7	(5.1)	6.8	(6.5)	1.06***	1.04	1.08	1.12***	1.09	1.15	1.06***	1.03	1.09
Avoidance (C)	1.5	(2.3)	2.2	(2.3)	3.0	(2.8)	1.13***	1.09	1.18	1.25***	1.18	1.33	1.11**	1.03	1.18
Negative cognitions and mood (D)	5.2	(7.0)	8.3	(8.1)	11.0	(8.7)	1.05***	1.04	1.07	1.09***	1.07	1.11	1.04**	1.01	1.06
Arousal (E)	5.0	(5.7)	7.4	(6.2)	10.4	(7.3)	1.06***	1.05	1.08	1.13***	1.10	1.16	1.06***	1.03	1.09

Note: *** = p < 0.001, ** = p < 0.01, * = p < 0.05. All models control for socio-demographic and service-related characteristics.

**Table 5:** Negative binomial regression model predicting gambling total score.

	Step 1			Step 2			Step 3		
	IRR	IRR	LB	UB	IRR	IRR	LB	UB	IRR
Trauma exposure									
Number of non-military lifetime traumas	1.10**	1.03	1.16	1.04	0.98	1.10	1.04	0.98	1.10
Number of traumatic deployment experiences	1.03	0.97	1.10	1.00	0.93	1.07	0.99	0.92	1.05
Depression									
PHQ-9 total score	-	-	-	1.10***	1.07	1.12	1.07***	1.03	1.11
PCL-5 PTSD subscales									
Re-experiencing (B)	-	-	-	-	-	-	0.99	0.93	1.05
Avoidance (C)	-	-	-	-	-	-	1.02	0.90	1.14
Negative cognitions and mood (D)	-	-	-	-	-	-	0.97	0.93	1.01
Arousal (E)	-	-	-	-	-	-	1.08*	1.01	1.14

Note:

*** = p < 0.001

** = p < 0.01

* = p < 0.05

All models control for socio-demographic and service-related characteristics.

## Discussion

Veterans who have recently left the military are vulnerable to the development of psychiatric disorders, particularly as they include a significant subgroup who have been medically discharged [26]. While much is known about alcohol abuse, PTSD, depression, anxiety, and other common disorders, there is very little research on gambling problems in veteran and military populations. This is the first comprehensive study of gambling problems and associated help-seeking behaviours in recently transitioned veterans, which also increases understanding of relationships involving trauma exposure. The results indicated that 13.4% of recently transitioned ADF veterans reported at least some gambling problems across a continuum of severity. This includes 4.6% of veterans that indicated clinically significant levels of problem gambling (PGSI ≥ 5), and 8.8% that reported sub-clinical levels of at-risk gambling (PGSI 1–4). This combined rate of 13.4% is higher than general civilian population surveys, which report a combined rate of 7.9% in Australia [27] The findings are also consistent with a previous study of Australian military personnel which reported that gambling problems are a significant issue for military population [3].

Time since transition was not associated with gambling problems, which contrasts with trajectories seen in other psychiatric disorders that often exhibit increases with time since transition [1]. These contrasting patterns of association with time since transition may suggest that gambling problems and related risk-taking behaviours precede the development of many other mental health problems among some transitioned members, and may act as an ‘up stream’ antecedent of such mental health problems, and accordingly comprise potential targets for early intervention and prevention. This proposal is consistent with civilian longitudinal research that shows gambling problems have a bi-directional effect on the development and maintenance of mental health problems [28]. Despite being common, however, the current results also show that veterans with problem gambling seek help typically for anxiety or depression, functional impairment, or other reasons such as chronic pain, rather than for gambling. Similarly, veterans with at risk gambling also reported having sought care primarily for anxiety or depression symptoms, as well as functional impairment. These findings are consistent with the broader literature indicating that help-seeking rates in civilians with problem gambling are very low [29], and indicate there is a need for strategies to increase help-seeking for gambling amongst veteran and military populations. Given that gambling problems are encountered more regularly in treatment for other conditions, identification strategies and responses must be situated in such settings.

Evidence for the relationship between trauma and gambling problems is emerging, and is critically important in military and veteran populations where trauma exposure is common [10]. Our results show that while military-related and non-military traumatic events, as well as PTSD symptoms and depression, all had associations with gambling problems, it is arousal and dysphoric-related affect factors that may explain the relationship between trauma and gambling problems. Controlling for socio-demographic, service-related characteristics, and trauma exposure, both depression symptoms and PTSD sub-scale hyper arousal scores were significant predictors of gambling problems, while non-military traumatic events were no longer significant when controlling for post-traumatic psychopathology. Consistent with previous research proposing that the relationship between trauma and gambling problems is driven by arousal and negative mood states [13], our findings indicate that arousal-related aspects of posttraumatic psychopathology are related to gambling problems. Of note, military-related traumatic experience had no unique relationship with gambling problems, which has particular relevance given the extent to which military and veteran populations experience deployment-related trauma. Our findings complement a smaller study that also found no associations between combat exposure and gambling problems [7].

### Limitations

The current study was characterised by notable strengths including large sample size and use of a range of validated scales with strong psychometric properties. However, the findings should also be viewed in the context of limitations. Firstly, the response rate was 18%, which is low, meaning results may not generalise to all transitioned veterans. The analyses were based on cross-sectional data and do not provide evidence of processes that unfold over time, including the likely directionality of associations. As such, the causal precedence of gambling problems over mental health and wellbeing indicators, while assumed by regression analyses, cannot be established using this cross-sectional data. Consistent with other literature, these gambling problems and associated risk factors explored here are likely to mutually reinforce each other. Furthermore, mode of gambling was not measured, with electronic gaming machines (EGMs) being associated with the largest gambling expenditure, while on-line and sports betting in younger males rapidly increasing in expenditure [28], and may be relevant to vulnerable sub-groups identified here. Finally, the data was captured in 2015 and may not accurately reflect current prevalence rates of gambling among current and ex-serving ADF members.

### Conclusions

Veterans are in a period of vulnerability during transition out of military service, and harms associated with gambling problems as an arousal/dysphoria management strategy may be significantly exacerbated during this period of transition. High rates of gambling problems indicate that addressing these addictive behaviours should be a key priority, with recently transitioned veterans particularly vulnerable. There is a need for universal prevention strategies for gambling problems in veterans that will cut across those within and outside veteran focused services. Such prevention strategies require consideration of gambling environments in Australia, and factors such as the accessibility of online gambling services and land-based gambling venues for transitioned military personnel. Identification and treatment for gambling problems need to consider underlying trauma histories and the high co-morbidity of other posttraumatic mental health problems, as well as the potential role for coping style. The findings here indicate there are barriers, either veteran and/or service focused to help-seeking for gambling problems that need addressing. Future research should consider further differences and similarities between trauma-exposed populations and gambling problems to improve intervention approaches.

## References

[1] Van Hooff M, Lawrence-Wood E, Hodson S, et al. *Mental Health Prevalence*, *Mental Health and Wellbeing Transition Study*. Canberra: the Department of Defence and the Department of Veterans’ Affairs;2018.

[2] Sharp M-L, Fear NT, Rona RJ, et al. Stigma as a barrier to seeking health care among military personnel with mental health problems. *Epidemiologic reviews*. 2015;37(1):144–162. doi: 10.1093/epirev/mxu012 25595168

[3] Hansen C, McFarlane A, Iannos M, et al. Psychosocial factors associated with psychological distress and functional difficulties in recently transitioned and current serving regular Australian Defence Force members. *Psychiatry research*. 2020;286:112860. doi: 10.1016/j.psychres.2020.112860 32065981

[4] Cowlishaw S, Metcalf O, Lawrence-Wood E, et al. Gambling problems among military personnel after deployment. *Journal of Psychiatric Research*. 2020;131:47–53. doi: 10.1016/j.jpsychires.2020.07.035 32920277

[5] Stefanovics EA, Potenza MN, Pietrzak RH. Gambling in a national US veteran population: Prevalence, socio-demographics, and psychiatric comorbidities. *Journal of gambling studies*. 2017;33(4):1099–1120. doi: 10.1007/s10899-017-9678-2 28293767

[6] Whiting SW, Potenza MN, Park CL, McKee SA, Mazure CM, Hoff RA. Investigating veterans’ pre-, peri-, and post-deployment experiences as potential risk factors for problem gambling. *Journal of Behavioral Addictions*. 2016;5(2):213–220. doi: 10.1556/2006.5.2016.027 27156377 PMC5387772

[7] Westermeyer J, Canive J, Garrard J, Thuras P, Thompson J. Lifetime prevalence of pathological gambling among American Indian and Hispanic American veterans. *American Journal of Public Health*. 2005;95(5):860–866. doi: 10.2105/AJPH.2003.023770 15855466 PMC1449269

[8] Dighton G, Roberts E, Hoon AE, Dymond S. Gambling problems and the impact of family in UK armed forces veterans. *Journal of behavioral addictions*. 2018;7(2):355–365. doi: 10.1556/2006.7.2018.25 29739238 PMC6174607

[9] Biddle D, Hawthorne G, Forbes D, Coman G. Problem gambling in Australian PTSD treatment‐seeking veterans. *Journal of Traumatic Stress*: *Official Publication of The International Society for Traumatic Stress Studies*. 2005;18(6):759–767. 10.1002/jts.20084 16382440

[10] Forbes D, Pedlar D, Adler AB, et al. Treatment of military-related post-traumatic stress disorder: challenges, innovations, and the way forward. *International Review of Psychiatry*. 2019;31(1):95–110. doi: 10.1080/09540261.2019.1595545 31043106

[11] Etuk R, Shirk SD, Grubbs J, Kraus SW. Gambling Problems in US Military Veterans. *Current Addiction Reports*. 2020:1–19.

[12] Scherrer JF, Xian H, Kapp JMK, et al. Association between exposure to childhood and lifetime traumatic events and lifetime pathological gambling in a twin cohort. *The Journal of nervous and mental disease*. 2007;195(1):72–78. doi: 10.1097/01.nmd.0000252384.20382.e9 17220743

[13] Roberts A, Sharman S, Coid J, et al. Gambling and negative life events in a nationally representative sample of UK men. *Addictive behaviors*. 2017;75:95–102. doi: 10.1016/j.addbeh.2017.07.002 28715699

[14] Sharpe L, Tarrier N, Schotte D, Spence SH. The role of autonomic arousal in problem gambling. *Addiction*. 1995;90(11):1529–1540. doi: 10.1046/j.1360-0443.1995.9011152911.x 8528038

[15] Baudinet J, Blaszczynski A. Arousal and gambling mode preference: A review of the literature. *Journal of gambling studies*. 2013;29(2):343–358. doi: 10.1007/s10899-012-9304-2 22484996

[16] Clancy CP, Graybeal A, Tompson WP, et al. Lifetime Trauma Exposure in Veterans With Military-Related Posttraumatic Stress Disorder: Association With Current Symptomatology.(CME). *Journal of Clinical Psychiatry*. 2006;67(9):1346–1353. doi: 10.4088/jcp.v67n0904 17017820

[17] Ferris JA, Wynne HJ. *The Canadian problem gambling index*. Canadian Centre on Substance Abuse Ottawa, ON; 2001.

[18] Williams RJ, Volberg RA. The classification accuracy of four problem gambling assessment instruments in population research. *International Gambling Studies*. 2014;14(1):15–28.

[19] Cowlishaw S, Gale L, Gregory A, McCambridge J, Kessler D. Gambling problems among patients in primary care: a cross-sectional study of general practices. *Br J Gen Pract*. Apr 2017;67(657):e274–e279. doi: 10.3399/bjgp17X689905 28289016 PMC5565808

[20] Cowlishaw S, Little J, Sbissa A, et al. Prevalence and implications of gambling problems among firefighters. *Addictive Behaviors*. 2020:106326. doi: 10.1016/j.addbeh.2020.106326 32004832

[21] Kroenke K, Spitzer RL, Williams JB. The PHQ-9: validity of a brief depression severity measure. *J Gen Intern Med*. Sep 2001;16(9):606–613. doi: 10.1046/j.1525-1497.2001.016009606.x 11556941 PMC1495268

[22] Weathers FW, Litz BT, Keane TM, Palmieri PA, Marx BP, Schnurr PP. *The PTSD Checklist for DSM-5 (PCL-5)*. Scale available from the National Center for PTSD at www.ptsd.va.gov. 2013.

[23] Blevins CA, Weathers FW, Davis MT, Witte TK, Domino JL. The Posttraumatic Stress Disorder Checklist for DSM-5 (PCL-5): Development and Initial Psychometric Evaluation. *Journal of traumatic stress*. Dec 2015;28(6):489–498. doi: 10.1002/jts.22059 26606250

[24] Haro JM, Arbabzadeh-Bouchez S, Brugha TS, et al. Concordance of the Composite International Diagnostic Interview Version 3.0 (CIDI 3.0) with standardized clinical assessments in the WHO World Mental Health Surveys. *International Journal of Methods in Psychiatric Research*. 2006;15(4):167–180. doi: 10.1002/mpr.196 17266013 PMC6878271

[25] Dobson A, Treloar S, Zheng W, et al. *The Middle East Area of Operations (MEAO) Health Study: Census Study Report*. Brisbane, Australia: The University of Queensland, Centre for Military and Veterans Health.; 2012.

[26] McFarlane A, Hodson S.E, Hooff MV, Davies C. Mental health in the Australian Defence Force: 2010 ADF Mental Health and Wellbeing Study: Full report. Department of Defence, Canberra 2011.

[27] Armstrong A, Carroll M. Gambling activity in Australia. *Findings from wave*. 2017;15.

[28] Hartmann M, Blaszczynski A. The longitudinal relationships between psychiatric disorders and gambling disorders. *International Journal of Mental Health and Addiction*. 2018;16(1):16–44.

[29] Loy JK, Grüne B, Braun B, Samuelsson E, Kraus L. Help-seeking behaviour of problem gamblers: a narrative review. *Sucht*. 2018;64(5–6):259–272.

